# Editorial: New techniques in microbiome research

**DOI:** 10.3389/fcimb.2023.1158392

**Published:** 2023-03-07

**Authors:** Tao Lin

**Affiliations:** Department of Molecular Virology and Microbiology, Alkek Center for Metagenomics and Microbiome Research, Baylor College of Medicine, Houston, TX, United States

**Keywords:** microbiome, human health and diseases, new techniques, meta-omics, microbiome Koch’s postulates

## Microbiome research of the past decades

The microbiota is the total collection of microbes that live inside and on our bodies, and the genes they encode are collectively known as the microbiome. Studies have described the roles of the microbiota in host health and a variety of disease states. Microbiome and meta-omics analysis of athletes have found a performance-enhancing microbe, specific gut bacteria can produce tastier cow milk, and centenarians have different gut microbiome that generate unique secondary bile acids. One of the most interesting studies was that the gut microbiota is associated with SARS-CoV-2 virus load and COVID-19 severity.

The second phase of the Human Microbiome Project has provided insights into Inflammatory Bowel Disease, Type 2 Diabetes, and premature birth. The knowledge, technology, and resources obtained from phases 1 and 2 of the Human Microbiome Project will be applied to disease diagnosis and treatment in the future. Studies on the mechanism of host-microbiota interactions present a formidable challenge. Analyses of host-microbiota interactions have provided mechanistic insights into the complex interplay between the host and the microbiota. These data will lead to new opportunities for diagnosis and treatment of a variety of human and animal diseases.

## Microbiome and human health and diseases

Microbiome research is driven by an interest in the mechanism of how the microbiota impacts and responds to disease as well as uncover the mechanisms behind the diseases. Microbiome and metagenomics studies have confirmed the importance of the microbiota to human health and diseases. To date, only a small percentage of the bacteria that comprise the human microbiota have been isolated, identified, and studied because the required growth conditions cannot be reproduced in the laboratory. However, recent technological advances in next-generation sequencing (NGS) and metagenomics have made it feasible to analyze the entire human microbiome. Improvements in technology have made it possible to study host-microbiota interactions. The application of cutting-edge technologies, such as *in vivo* monoclonization and functional assays in specific pathogen free (SPF) and germ-free (GF) mouse models, *in vitro* co-cultures of the anaerobic microbiome and aerobic host tissues, and ‘meta-omics’-related research (such as metagenomics, transcriptomics, metabolomics, proteomics, and spatial resolution) have provided major insights into the complexity of relationships between the host and microbiome as well as the mechanisms of the functional roles of the microbiota and individual bacteria on host health and disease. The anaerobic intestine-on-a-chip system enables complex microbiota co-cultures. This tool could be used to study the mechanisms of host-microbiota interactions and facilitate discovery and development of microbiome-related therapeutics.

## What is next for the human microbiome?

**Figure 1:**
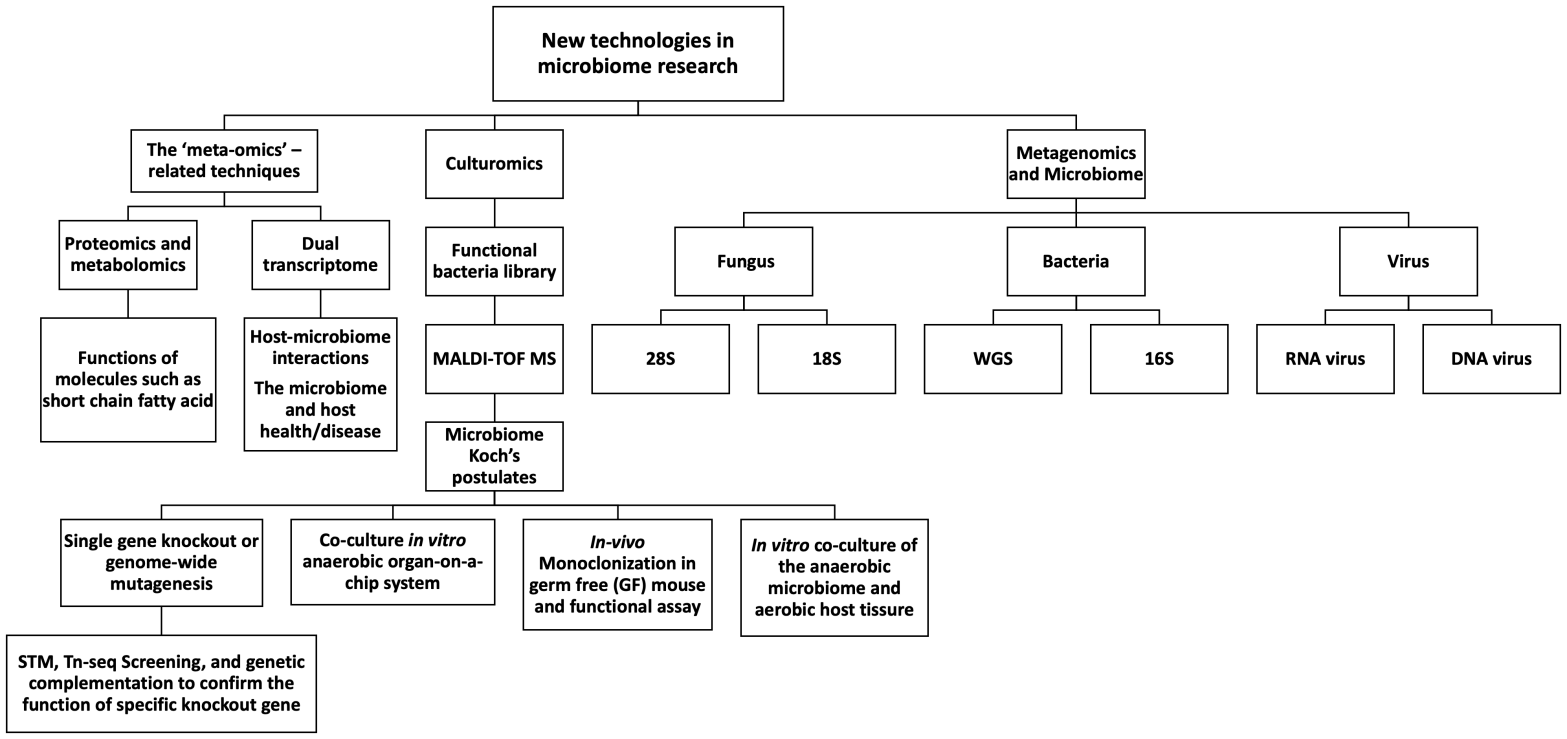
New techniques in microbiome research.

An increased understanding of the microbiota and its functional roles in host health and diseases is fundamental to the discovery and implementation of strategies for disease diagnosis and treatment, an example being the influence of probiotics on health, bacteria, bacteria products, or microbiota for potential improvement in the treatments of cancers and other diseases. Medical insights will flow from dissecting host-microbe interactions using state-of-the-art ‘meta-omics’-related research, image technologies, *in-vitro* co-culture systems, and *in-vivo* monoclonization of bacteria, virus, or other microbes in SPF and GF mice. Organ-on-a-chip systems are another tool to study host-microbiota interactions. High throughput genome-wide mutagenesis, such as transposon mutagenesis, Signature Tagged Mutagenesis (STM), transposon insertion sequencing (Tn-seq), molecular and microbiome Koch’s postulates, and more will be utilized to study the host-microbiome interactions and functional analysis of the microbiome (**Figure 1**).

## Successful collections of studies in this Research Topic

Some key results and techniques were published in this Research Topic, including 7 original research, 4 reviews, and one commentary. These articles reflect recent developments in science and technology in microbiome research, including the microbiome and diseases, microbiome and neurodegenerative disorders (Ceppa et al., 2020), vaginal and endometrial microbiota and female infertility (Riganelli et al., 2020), irradiation-induced intestinal damage recovery by *Lactobacillus acidophilus* (Sittipo et al., 2020), and roles of microbiota in Polycystic Ovary Syndrome (Dong et al., 2021).

Other important technological advances include host-microbiome interactions in a single cell (Sharma and Thaiss, 2020), discovering novel metabolites in the microbiome (Couvillion et al., 2020), breath biopsy and volatile organic compounds for diagnosis of infectious diseases (Belizario et al., 2020), and high throughput culture and rapid identification of the microbiome (Naud et al., 2020).

One data analysis article (Galazzo et al., 2020) analyzes quantitative microbiome profiling (QMP) using cell-based flow cytometry (Vandeputte et al., 2017) and quantitative PCR (qPCR) (Jian et al., 2020). The cell-based flow cytometry article and its commentary using qPCR (Jian et al., 2020) brought the potential solutions and discussion for the key issue we faced in this field: how to calculate the absolute and relative abundance profiles. The compositional nature of microbiome datasets makes it challenging to identify microbial taxa. Galazzo et al. compared the QMP by integrating absolute quantification of microbial abundances into the next generation sequencing data. Both cell-based methods (flow cytometry) and molecular methods (qPCR) have been used to determine the absolute microbial abundances. The authors compared relative microbiome profiling to microbial cell counting using flow cytometry, flow cytometry combined with the microbial composition of intact cells, and molecular-based quantification using qPCR targeting the 16S rRNA gene. Although qPCR and flow cytometry both resulted in accurate and strongly correlated results when quantifying the bacterial abundance of a mock community of bacterial cells, the two methods resulted in highly divergent QMPs. They demonstrate variability arising from different QMP methods. However, Jian et al. think the presented results are insufficient to make a conclusion that flow cytometry is a preferable method for QMP (Jian et al., 2020). Importantly, a conceptual pitfall in cell based QMP may lead to intractable biases.

Another article reviewed research advances on the roles of the human gut microbiota in neurodegenerative disorders and current engineered tools. Recent studies have confirmed the links between the gut microbiota and neurodegenerative disorders such as Alzheimer’s and Parkinson’s diseases. Most microbes in the gut might influence other organs through their metabolites. The role of gut microbiota in the pathogenesis of the human brain remains unclear. The gut-microbiota-brain crosstalk might involve neurodegeneration, but the mechanism has not been elucidated due to the limitations of available research tools. New technologies might help to understand the causative role of gut microbes in neurodegeneration. Therefore, this paper reviews the recent advances in the study of the microbiota-gut-brain axis in the field of neurodegenerative disorders to identify specific microbial signaling pathways and characterize advanced engineered tools to study the interactions between human cells and gut bacteria.

Microbes are the most prevalent members in the human gut. However, understanding their functional roles in human health and disease has been challenging due to the lack of molecular and cellular analysis tools. One of the recent advancements is single-cell analysis through genomics, transcriptomics, and spatial resolution. These techniques have been applied to microbiome analysis and have significantly advanced our understanding of host responses, microbial composition and diversity, and host-microbe interactions. One review in this Research Topic highlights the emerging single-cell analysis technologies that provide insights into microbe composition and diversity, the impact of microbes on host response, and host-microbiota interactions. The single-cell analysis sheds light on better understanding host-microbiota interactions and disease diagnosis and treatment (Sharma and Thaiss, 2020).

One of the original research articles examines the structural variations of vaginal and endometrial microbiota of females with infertility. The preliminary results improve our knowledge of the genitourinary microbiota and highlight the putative relationship between vaginal/endometrial microbiota and reproductive success. The uterus was considered to be a sterile environment for years. However, this study indicated that microbiota in the reproductive tract (RT) of asymptomatic and infertile women might be associated with implantation failure. The vaginal microbiota of pregnant women exhibited a lactobacilli-dominant habitat, while lactobacilli were exclusively detected in the group that displayed unsuccessful *in vitro* fertilization.

## Conclusion

Two decades ago, research on the human microbiome was a fledgling field. Now, it is a flourishing and attractive research area. We are not able to collect all the attractive papers in the first volume of this Research Topic. However, given the success of Volume I of this Research Topic, we are pleased to announce the launch of Volume II of *New Techniques in Microbiome Research*. We hope to see more attractive papers and emerging techniques soon.

## Author contributions

The author confirms being the sole contributor of this work and has approved it for publication.

